# Proximate, Physicochemical, Functional and Sensory Responses of Garlic Paste Formulations Combining *Allium sativum* and *Allium ampeloprasum*

**DOI:** 10.3390/foods15183203

**Published:** 2026-09-10

**Authors:** Nelson Blanco-Rodríguez, Atharva Rosa-de la Cruz, Julio Mejía-Brea, Esclaudys Pérez-González, Yulisa Alcántara-Marte, Yanilka Alcántara-De Tejada

**Affiliations:** 1 Department of Agronomy, Universidad ISA, Av. Presidente Antonio Guzmán Fernández km 5 1/2, Santiago de los Caballeros 51011, Dominican Republic; 20210141@isa.edu.do (N.B.-R.); eperez@isa.edu.do (E.P.-G.); 2 Institute of Innovation in Biotechnology and Industry (IIBI), Av. Olof Palme at José Núñez de Cáceres, Santo Domingo 10134, Dominican Republic; aveda@iibi.gob.do (A.R.-d.l.C.); jmejia@iibi.gob.do (J.M.-B.); 3 Department of Food Technology, Universidad ISA, Av. Presidente Antonio Guzmán Fernández km 5 1/2, Santiago de los Caballeros 51011, Dominican Republic; yualcantara@isa.edu.do

**Keywords:** *Allium sativum*, *Allium ampeloprasum*, garlic paste formulation, genotype combinations, sensory acceptance

## Abstract

The optimization of agro-industrial products represents a key strategy to enhance their technological quality, stability, and market acceptance. The aim of this study was to evaluate the effect of different proportions of *Allium ampeloprasum* (elephant garlic, E) and *Allium sativum* genotypes Roselló 1 (R) and Taiwan 5 (T) on the nutritional, physicochemical, functional, rheological, and sensory properties of garlic paste formulations. Binary mixtures were prepared at E:*A. sativum* ratios of 25:75, 50:50, and 75:25 for each genotype, together with pastes containing 100% of each garlic material, resulting in nine formulations. The results showed that mixture proportion and *A. sativum* genotype influenced several product attributes. Among the mixtures, E_25_T_75_ showed the highest protein content (4.37%), whereas E_50_T_50_ exhibited the highest antioxidant capacity (41.71 ± 0.94 µmol TE 100 g^−1^). Syneresis was significantly affected by formulation, storage time, and their interaction (*p* < 0.001), demonstrating formulation-specific changes during the 21-day storage period. Quadratic Scheffé mixture modeling showed a significant positive deviation from additivity for antioxidant capacity in the E + T system (β_12_ = 19.0, *p* = 0.019), whereas the E + R system was consistent with additive behavior (β_12_ = −10.2, *p* = 0.455). In the sensory analysis, the first two principal components explained 83.5% of the total variability, with Dim1 (49.4%) associated mainly with color and texture and Dim2 (34.1%) with flavor and aroma. Among the mixtures, E_75_T_25_ showed the highest mean aroma score (3.57), whereas E_75_R_25_ and E_25_T_75_ showed the highest mean flavor scores (3.45 and 3.48, respectively). Overall, sensory responses varied according to both mixture proportion and *A. sativum* genotype, with no single formulation maximizing all evaluated attributes. These findings demonstrate that combining *A. ampeloprasum* with different *A. sativum* genotypes generates formulation-specific technological, functional, and sensory responses.

## 1. Introduction

Continuous improvement in food quality, encompassing nutritional, chemical, sensory, and functional aspects, has been a cornerstone of modern food research. Within this context, common garlic (*Allium sativum*) is a highly important ingredient in global cuisine and medicine because of its culinary versatility and notable health benefits, and it has therefore been the subject of numerous studies [1,2,3]. As an ingredient, it is valued in the food industry for its nutritional value and its ability to improve sensory profiles and extend food shelf life [4,5], providing a healthy and effective alternative to synthetic preservatives [6]. The incorporation of garlic into food innovations continues to position it as an essential ingredient in cooking and in the development of functional products that respond to current health and wellness trends [7,8]. In the Dominican Republic, as in many regions, garlic production is primarily destined for fresh consumption. This market structure limits the availability of raw material for agro-industrial processing and creates vulnerability in the supply chain, as competition between fresh and processed markets intensifies domestic price volatility [9]. To increase supply and diversify processing opportunities, elephant garlic (*Allium ampeloprasum* L.) represents a promising alternative to common garlic, particularly because of its higher productivity [10]. Its considerably larger bulbs and greater biomass production than *A. sativum* further support its potential for agro-industrial applications [11,12]. Although these two species differ in their physicochemical and structural traits [13,14], these differences may provide functional advantages for the development of new products with distinct sensory and nutritional properties [15,16,17]. Garlic germplasm cultivated in the Dominican Republic exhibits marked variability in chemical and bioactive profiles, including total fat, allicin, phenolics, flavonoids, tannins, and antioxidant capacity. A previous characterization of garlic germplasm cultivated in Constanza demonstrated that Taiwan 05 showed particularly high allicin concentration and antioxidant capacity, whereas Morado Rosello #1 exhibited the highest antioxidant capacity among the genotypes evaluated [18]. Such genotype-dependent variation indicates that the genetic background of *A. sativum* may contribute to differences in the quality attributes of garlic-derived products. Combining *A. sativum* and *A. ampeloprasum* may therefore provide complementary characteristics for garlic paste formulation, depending on the proportion of each species and the genotype of the *A. sativum* component. Common garlic provides a high concentration of organosulfur compounds associated with its characteristic aroma, flavor, and bioactivity [1,8], whereas elephant garlic contributes a softer texture and higher biomass yield that may be advantageous for paste preparation [19]. Rather than assuming that binary mixtures are universally superior to pure formulations, evaluating these species at different proportions provides an opportunity to determine how compositional changes affect the nutritional, physicochemical, rheological, functional, and sensory properties of the resulting paste. Despite the documented diversity among garlic genotypes and the distinct characteristics of *A. ampeloprasum* and *A. sativum*, limited information is available on their combined use at different proportions in garlic paste formulations, particularly across different *A. sativum* genetic backgrounds. It remains unclear whether changing the proportion of elephant garlic produces consistent responses across product attributes or whether the response depends on the *A. sativum* genotype and the attribute evaluated. To address this knowledge gap, the objective of this study was to evaluate the effects of different mixture proportions of *A. sativum* and *A. ampeloprasum* on the nutritional, physicochemical, functional, rheological, and sensory properties of garlic pastes. The study specifically examined whether these attributes varied according to mixture proportion and *A. sativum* genotype. We hypothesized that changing the proportion of *A. ampeloprasum* and the genetic background of *A. sativum* would differentially modify the properties of the resulting pastes, and that the responses would vary among attributes and could deviate from the additive behavior expected from the individual components.

## 2. Materials and Methods

**Study location:** The study was conducted in the laboratories of the Department of Food Technology and the Plant Protection Laboratory of the Department of Agronomy at Universidad ISA, both located at Avenida Presidente Antonio Guzmán Fernández, km 5½, La Herradura, Santiago de los Caballeros, Dominican Republic.

**Raw material:** The raw materials included elephant garlic (*Allium ampeloprasum* L.) and two *Allium sativum* genotypes designated Roselló 1 and Taiwan 5. All genotypes were supplied by the IDIAF Experimental Station in Constanza, Dominican Republic. Other ingredients and additives, including fine salt, oil, granulated sugar, acetic acid, sodium benzoate, citric acid, and carboxymethylcellulose (CMC), were used according to the garlic paste formulation. Garlic was the only component that varied among formulations; the same total amount was used, but the genotype proportions differed.

**Description of garlic genotype biomass formulations:** Different proportions of the two garlic species were formulated, with elephant garlic used alone or combined with Roselló 1 or Taiwan 5, as shown in Table 1. A total of nine formulations were evaluated, including the three pure-genotype formulations and six binary mixtures. The 25:75, 50:50, and 75:25 proportions were selected as an exploratory substitution gradient to characterize the response of the paste across different relative contributions of *A. ampeloprasum* and each *A. sativum* genotype, covering low, intermediate, and high proportions of elephant garlic without assuming a priori an optimal formulation. In each formulation, the total amount of garlic was kept constant, while only the relative proportion of *A. ampeloprasum* and the *A. sativum* genotype was varied. Three independent paste lots (individual production batches) were prepared for each formulation and considered the independent experimental replicates for the analytical evaluations.

### 2.1. Garlic Paste Processing Method

**Figure 1:**
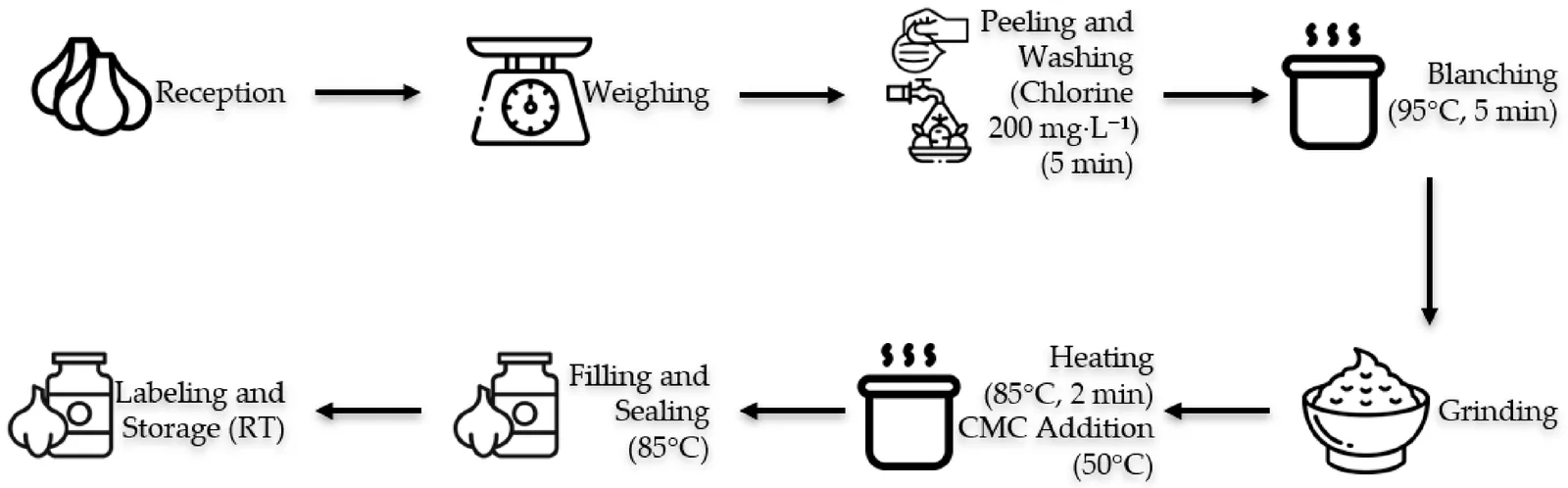
Flow Diagram of the Garlic Paste Production Process.

Garlic paste processing began with receiving and weighing the bulbs, followed by peeling and washing in a chlorine solution (200 mg·L^−1^) for 5 min. The cloves were then blanched at 95 °C for 5 min to soften the tissue and facilitate paste production. After blanching, the garlic was cooled to approximately 50 °C and ground in an industrial food blender (Model 61BL30, Waring Commercial, Torrington, CT, USA) until a homogeneous mass was obtained. Once the paste had formed, the additional ingredients were incorporated according to the established formulation, and uniform distribution was ensured by continuous mixing. At this same stage (≈50 °C), carboxymethylcellulose (CMC) was added, after which the final mixture was heated at 85 °C for 2 min for thermal stabilization. Finally, the hot paste was filled and sealed at 85 °C, and the containers were labeled and stored at room temperature, as shown in Figure 1. The quantitative formulation of the paste was identical across treatments except for the relative proportion of the garlic genotypes.

For each 450 g production batch, the formulation contained 396.0 g of garlic biomass (88.00%), 18.0 g of refined vegetable oil (4.00%), 18.0 g of sodium chloride (4.00%), 10.915 g of refined sugar (2.41%), 6.5 g of 5% acetic acid solution (1.50%), 0.225 g of sodium benzoate (0.05%), 0.09 g of citric acid (0.02%), and 0.09 g of carboxymethylcellulose (0.02%). The garlic component within the 396.0 g total was distributed according to the proportions specified in Table 1.

### 2.2. Data Collection

Three independent paste lots were prepared for each formulation. The analytical determinations were performed using samples from these independent lots, and the three lots constituted the experimental replicates. Where multiple readings or determinations were obtained from the same lot, these measurements were averaged before statistical analysis to avoid treating repeated measurements within a lot as independent experimental replicates.

### 2.3. Nutritional Analyses

**Protein:** The Kjeldahl method was followed with minor modifications, using TiO_2_ as the catalyst to improve digestion efficiency instead of CuSO_4_. Total nitrogen was converted to crude protein using a conversion factor of 6.25 (*N* × 6.25). The result was expressed as mean protein percentage.

**Total fat:** The Soxhlet method was used with petroleum ether as the extraction solvent. Between 2 and 5 g of the previously dried sample was weighed and extracted in the Soxhlet apparatus for 6 h.

**Ash:** Ash content was determined by incineration in a muffle furnace (Thermolyne^TM^ FB1410M, Thermo Fisher Scientific, Asheville, NC, USA) at 550 °C, following the AOAC (2007) protocol, to quantify inorganic components and determine the mineral content of the sample.

**Moisture:** Moisture content was estimated gravimetrically by drying the sample at 65 °C to constant weight.

Each of these variables was expressed as a percentage.

### 2.4. Physicochemical Analyses

**pH:** Hydrogen ion potential (pH) was determined according to AOAC method 981.12 using a 1:10 sample-to-water dilution (*m*/*v*), which was stirred for 5 min. The solution was then filtered, and the pH of the filtrate was measured using a Basic pH meter 20 (Hach Company, Loveland, CO, USA). Results were expressed in pH units.

**Total soluble solids (TSS):** TSS was determined by refractometry according to ISO 2173:2003 [20]. One or two drops of the sample were placed on the prism of a portable refractometer (RHB-32, China) while maintaining the temperature constant at 20 °C. Results were expressed in degrees Brix (°Brix), indicating the soluble solids content of the sample.

**Color:** Color was determined using a CS-10 portable Colorimeter (CHNSpec Technology Co., Ltd., Hangzhou, China) by recording lightness (*L*), red–green axis (*a*), and yellow–blue axis (*b*) values. The garlic paste sample was placed in a sterile polystyrene Petri dish and covered, and the colorimeter was positioned over the dish to obtain *L*, *a*, and *b* readings, which were averaged across replicates.

**Syneresis:** This variable was measured according to Sodhi and Singh et al. (2005) [21], with minor modifications. For each independent production batch, 50 g samples were heated at 90 °C for 30 min, rapidly cooled, and stored at 4 °C. Syneresis was evaluated after 7, 14, and 21 days of storage by centrifugation at 4000 rpm for 5 min using a clinical centrifuge (C3303, VWR, Radnor, PA, USA). Syneresis was expressed as the percentage ratio of the mass of released fluid to the initial sample mass. Relative change between Days 7 and 21 was calculated for each independent batch as [(S7 − S21)/S7] × 100, where S7 and S21 represent syneresis at Days 7 and 21, respectively. Positive values indicate a decrease in syneresis during storage, whereas negative values indicate an increase.

### 2.5. Functional and Sensory Properties

**Antioxidant capacity:** Antioxidant capacity was determined using the 2,2-diphenyl-1-picrylhydrazyl (DPPH) free-radical method described by Brand-Williams et al. (1995) [22], with minor modifications. The DPPH radical solution was prepared by dissolving 2 mg of DPPH in 250 mL of 80% methanol and adjusting the solution to an absorbance of 1.000. The reaction mixture consisted of 50 µL of garlic paste extract and 1.5 mL of DPPH solution for 30 min. The reaction was conducted at room temperature and in total darkness, and absorbance was measured at 517 nm using a Genesys 50 UV–Vis spectrophotometer (Thermo Fisher Scientific, Madison, WI, USA). For quantification, a Trolox calibration curve covering the range of 0–800 μM was prepared in 80% methanol. The resulting linear calibration equation (y = −0.0004x + 0.9895; R^2^ = 0.9767) was used for quantification, and results were expressed as micromoles of Trolox equivalents per 100 g of sample (µmol TE·100 g^−1^).

**Sensory acceptance:** Sensory evaluation was conducted with 60 untrained student consumers from Universidad ISA following the general guidelines of ISO 11136:2014 [23]. Nine coded samples corresponding to the nine garlic paste formulations were presented for individual evaluation. Aroma, color, texture, and flavor were evaluated using the same structured five-point hedonic scale, ranging from 1 = dislike extremely to 5 = like very much. Water was provided as a palate cleanser between samples. Responses were recorded using the sensory evaluation form developed for the study. Specific demographic information on the participants and quantitative records of room temperature and illumination were not systematically recorded and therefore are not reported retrospectively.

### 2.6. Statistical Analysis and Mixture Modeling

Data were subjected to descriptive statistics and a one-way analysis of variance (ANOVA) to evaluate differences among the nine garlic paste formulations for variables measured at a single time point. When statistically significant differences were detected (*p* < 0.05), mean comparisons were conducted using Tukey’s HSD test at a 95% confidence level. These preliminary analyses were performed using Navure Professional+ 4.0.1 statistical software.

Because syneresis was measured repeatedly on the same independent production batches during storage, it was analyzed using a linear mixed-effects model. Formulation (nine levels), storage time (7, 14, and 21 days), and their interaction were included as fixed effects, whereas production batch was included as a random effect to account for repeated observations from the same experimental unit over time. Significance of fixed effects was evaluated using Type III tests with Satterthwaite approximation for degrees of freedom. Pairwise comparisons among formulations within each storage time were performed using estimated marginal means with Tukey adjustment for multiple comparisons. Statistical significance was established at *p* < 0.05. Relative change in syneresis between Days 7 and 21 was retained as a descriptive summary of temporal behavior rather than as the response variable for inferential testing.

To determine whether the response of a binary mixture deviated from the additive behavior expected from its individual components, a quadratic Scheffé mixture model was fitted separately for each binary system (*A. ampeloprasum* + Roselló 1 and *A. ampeloprasum* + Taiwan 5):

$$Y=\beta_{1}X_{1}+\beta_{2}X_{2}+\beta_{12}X_{1}X_{2}$$

where Y represents the response variable, X_1_ and X_2_ correspond to the relative proportions of the two garlic components in the mixture (X_1_ + X_2_ = 1), β_1_ and β_2_ are the coefficients associated with the pure components, and β12 represents the mixture interaction term. A statistically significant positive β_12_ coefficient (*p* < 0.05) was interpreted as a positive deviation from additivity, whereas a statistically significant negative β_12_ coefficient indicated a negative deviation from additivity. A non-significant β_12_ coefficient (*p* ≥ 0.05) was interpreted as behavior consistent with additivity. The practical interpretation of the deviation was based on the directionality of each response variable. For syneresis, separate Scheffé models were fitted at Days 7, 14, and 21, while temporal changes were evaluated using the mixed-effects model described above.

The model was fitted using the individual observations from the three independent experimental lots for each formulation. Thus, each formulation contributed *n* = 3 independent observations, and each binary system comprised five formulations and 15 observations in total (*N* = 15). Complete estimates of the mixture interaction coefficient (β_12_), associated significance tests, and interpretation of deviation from additivity for the evaluated responses are provided in Table S3.

Principal component analysis (PCA) was performed on the mean hedonic scores of the nine formulations for aroma, color, texture, and flavor to explore multivariate relationships among sensory attributes and formulation profiles. Variables were standardized prior to PCA.

Mixed-effects analysis, mixture modeling, and PCA were conducted using R software (version 4.6.0; R Core Team, 2026).

## 3. Results and Discussion

### 3.1. Nutritional Variables

The different garlic proportions comprising the pastes significantly affected the percentages of protein, fat, ash, and moisture (Table 2).

**Protein:** As shown in Table 2, the pastes prepared from the 100% *A. sativum* controls (both Roselló and Taiwan genotypes) had the highest protein percentages (4.77 and 4.67%, respectively) and were statistically equivalent. For this macronutrient, T100 had a value statistically similar to that of the E_25_T_75_ combination (4.37%). This variable appears to be influenced by the *Allium sativum* genotypes. Because garlic is not a primary protein source, its paste would be expected to contain a relatively low percentage of this nutritional component compared with other vegetables. This agrees with Zang et al. (2023) [24], who reported a protein content of 2.3% in garlic paste. Protein content is generally determined in fresh garlic [25], whereas this type of analysis is more limited in processed products such as garlic paste because processing methods may alter protein composition [26].

**Fat:** The fat content results for the different garlic paste formulations revealed significant differences among genotype combinations. Among the mixtures, E_25_T_75_ had the highest fat content (2.03%), followed by E_75_T_25_ (1.94%). In general, treatments containing higher proportions of the Taiwan genotype (T) had higher fat contents, suggesting a significant influence of this genotype on the lipid composition of the garlic pastes. Fresh garlic has a relatively low fat content. It has been reported to contain approximately 0.1 to 0.2 g of fat per 100 g of mass [27]. In fresh garlic, lipids are represented mainly by neutral lipids, glycolipids, and phospholipids, with a total content ranging from 0.31 to 0.34% [27,28]. Because edible oils are often incorporated during garlic paste processing to improve sensory and preservation properties, total fat content in the final product tends to increase relative to fresh garlic [29], as shown by the results in Table 2. Although several studies have addressed the lipid content of fresh garlic, research on the lipid profile of garlic paste is limited, leaving a gap in the literature regarding how processing and transformation of garlic into paste may affect its fat composition. The lipid content of garlic paste is of interest because of its potential health benefits and nutritional value. Garlic (*Allium sativum*) contains several lipid fractions that contribute to its overall lipid profile [30]. The low fat content of fresh garlic and, consequently, garlic paste may be associated with multiple health benefits, particularly regulation of the lipid profile and prevention of cardiovascular diseases [28].

**Ash:** Although the pastes combining garlic genotypes showed significant statistical differences for this variable, mixtures containing 50% or 75% elephant garlic showed a consistent increase in ash percentage. This increase is associated with the high ash content of the 100% elephant garlic paste, which reached 13.96% and was significantly higher than that of the other pastes in the study. These results suggest that even at moderate proportions, elephant garlic has a considerable effect on the mineral content of the pastes. In fresh garlic, ash content varies by genotype, ranging from 3.05% to 6.92% [31,32,33]. By comparison, garlic pastes contain approximately twice this amount because water reduction during processing concentrates minerals and because additional ingredients, such as salt, are included. In particular, elephant garlic, whose fresh-state ash content ranges from 2.59% to 3.14% [34], reached 13.96% in the E_100_ paste, demonstrating a substantial processing-related increase. Ash content in processed foods such as garlic paste (*A. ampeloprasum* and *A. sativum*) is highly important for evaluating nutritional quality because it reflects the concentration of minerals remaining after combustion of organic matter. This parameter is considered a direct measure of total mineral content and is an essential component of proximate food analysis [35]. In comparison, other processed condiments such as tomato paste (3–5%) and spices such as curry and cumin (up to 6%) have lower ash values [36,37].

**Moisture:** In this study, the evaluated garlic pastes had moisture contents above 60%, with statistically significant differences observed for this variable. Pastes containing a higher proportion of the Roselló genotype, such as E_25_R_75_ and E_50_R_50_, had lower moisture contents of 60.22% and 63.62%, respectively. Lower moisture levels may contribute to product stability; however, moisture content alone is insufficient to infer microbiological safety or shelf life. Moisture content is a key factor in garlic paste stability because high percentages may shorten product shelf life and increase susceptibility to microbial growth [24]. The moisture content of processed foods such as garlic paste is determined by factors including processing techniques, mechanical and thermal handling, and ingredient composition. Garlic naturally contains 58% to 66% water; these treatments may substantially reduce moisture, although products such as pastes generally retain high levels. Previous studies reported moisture contents of 67% to 69% in garlic pastes and garlic–ginger blends [38,39], consistent with the findings of the present study.

### 3.2. Physicochemical Properties

The physicochemical properties of the garlic pastes are presented in Table 3.

**Hydrogen ion potential (pH):** Garlic paste pH depends both on the initial content of acidic compounds in the genotypes used and on changes induced during processing and storage. The addition of both acetic and citric acids to the formulation, in the same amounts for each formulation, must also be considered. In this study, the evaluated combinations showed intermediate pH values ranging from 5.09 to 5.56, suggesting moderate acidity that could influence the microbiological and sensory stability of the final product. Prati et al. (2010) [40] evaluated garlic pastes prepared with different proportions of garlic and vinegar and reported pH values ranging from 3.53 to 4.10 depending on storage time and formulation, indicating that lower pH is common in products designed for greater stability. Carbonell-Barrachina et al. (2003) [41] suggested that a pH range of 4.0 to 4.5 is ideal for garlic pastes because it balances acidity, improves sensory acceptance, and ensures stability against microbial spoilage during storage. In this context, the pH values observed in this study were above the threshold considered optimal. Although this does not invalidate their functionality, it suggests that the formulation could be optimized to improve shelf life and potential product acceptance.

**Total soluble solids (TSS):** Mixtures containing the Roselló genotype showed greater variability in TSS values than formulations containing the Taiwan genotype, suggesting differences in the concentration of soluble solutes and potentially in system stability. Garlic paste typically has a TSS content of 30–35 °Brix [38], a range consistent with the values obtained in this study, in which the combined garlic formulations ranged from 30.53 °Brix to 34.53 °Brix. These variations may be attributed to genotypic differences affecting the composition of sugars, sulfur compounds, and other dissolved solutes [33]. Higher TSS content has been associated with better consistency and aqueous-phase stability in garlic pastes, which may positively influence sensory acceptance during storage [40,42].

**Figure 2:**
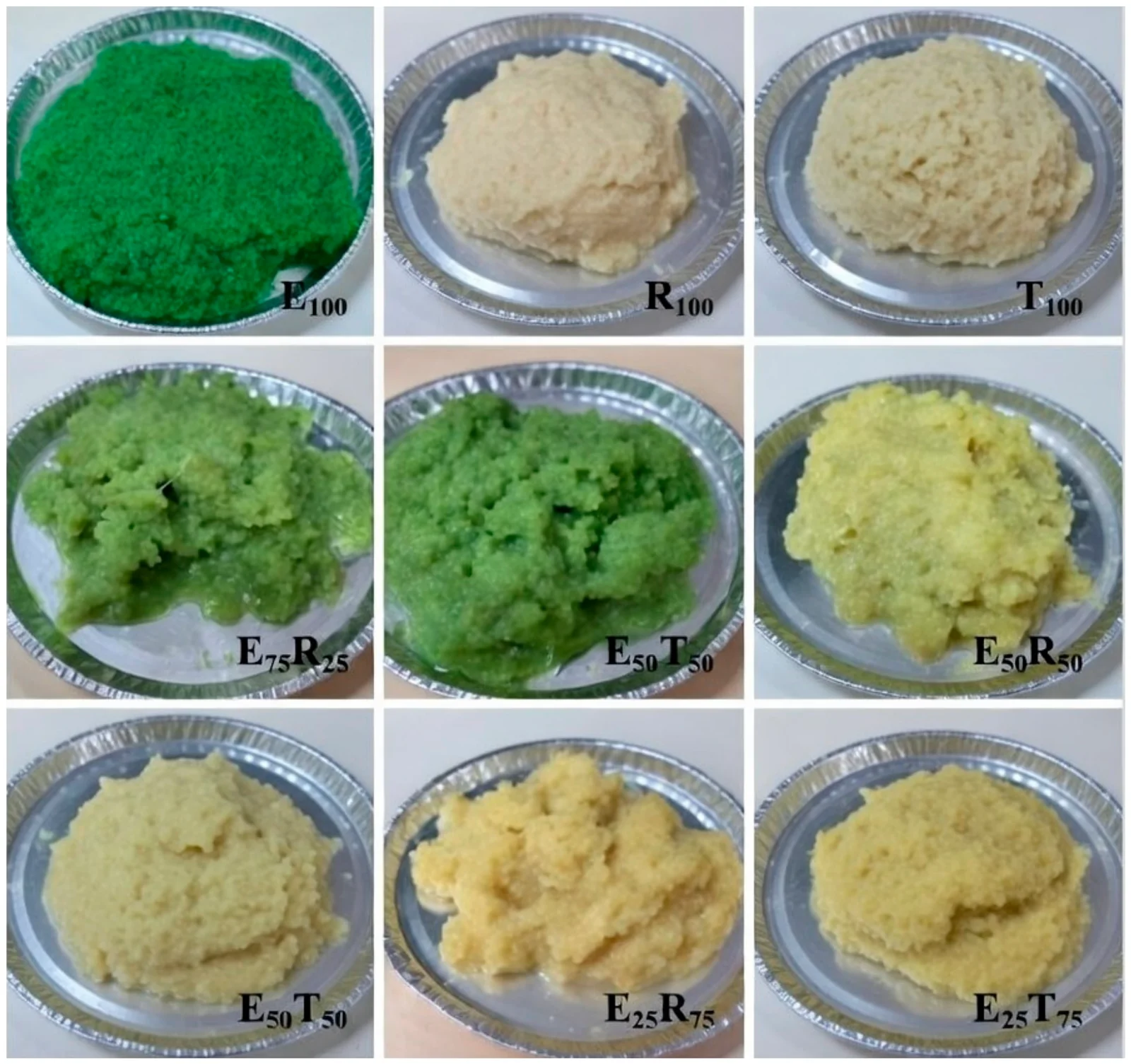
Representative images of garlic paste formulations under standardized lighting. Control samples (E_100_, R_100_, T_100_) and 25:75, 50:50, and 75:25 mixtures of *Allium ampeloprasum* (E) and the *Allium sativum* genotypes Roselló 1 (R) and Taiwan 5 (T) are shown.

**Color parameters (*L*, *a*, and *b*):** Combinations of elephant garlic (*A. ampeloprasum*) with *A. sativum* genotypes (Roselló and Taiwan) produced a range of paste colors that balanced lightness and green and yellow hues. In terms of lightness (*L*), combinations of *A. ampeloprasum* and *A. sativum*, particularly with the Taiwan genotype, produced lighter pastes. The E_50_T_50_ mixture had the highest *L* value (84.30) and was the lightest paste among the treatments studied. The E_75_T_25_ combination was also noteworthy because it maintained a balance between lightness and a moderate green hue, with *L* and *a* values of 66.63 and −22.50, respectively. According to the results, these combinations allow the color profile to be modified according to the proportions used, with elephant garlic contributing greater intensity of green and yellow tones, whereas mixtures with *A. sativum* provide more versatile lightness and chromatic balance options suitable for various products according to color and appearance requirements (Table 3, Figure 2).

The green color of garlic paste is caused mainly by biochemical reactions involving amino acids, leading to the formation of pyrrole-based compounds that impart a bluish-green color [43], and the conversion of sulfur compounds during processing and storage. The greater intensity of green color observed when elephant garlic (*A. ampeloprasum*) was present at a proportion of 50% or higher may be explained by differences in the composition of sulfur compounds and pigmentation metabolic pathways compared with common garlic (*A. sativum*). Although the latter contains higher concentrations of allicin and other cysteine sulfoxides [13], elephant garlic has a distinct profile that may include more reactive precursors for green pigment formation under certain conditions. Factors such as alkaline pH, which promotes interactions between sulfur compounds and amino acids, and greater activity of genetic and enzymatic pathways involved in pigment biosynthesis in *A. ampeloprasum* may intensify this phenomenon [44,45,46]. In addition, thermal processing and handling of elephant garlic may activate these pathways more efficiently, generating chromophoric pigments derived from sulfenates and amino acids such as cysteine and lysine [46].

The differences in coloration among the garlic paste formulations are illustrated in Figure 2. Because visual appearance can influence consumer perception and acceptance [47,48], these color differences may be relevant to the sensory characteristics of the formulations. However, the specific influence of green coloration on consumer acceptance or purchase intention was not evaluated in the present study. From a technological perspective, differently colored garlic pastes may have potential applications in formulated products such as sauces or seasonings [49], although such applications were not investigated here. Color changes in food products may also occur in association with oxidative processes, as lipid oxidation can generate compounds that affect color, odor, flavor, texture, and other quality attributes [50,51]. However, oxidative changes were not measured in the present study; therefore, lipid oxidation cannot be established as the cause of the coloration observed in these garlic paste formulations.

**Syneresis:** Syneresis was significantly affected by formulation (*F*(8, 18) = 4329.1, *p* < 0.001), storage time (*F*(2, 36) = 7920.5, *p* < 0.001), and their interaction (*F*(16, 36) = 2107.3, *p* < 0.001). The significant formulation × storage-time interaction indicated that changes in syneresis during storage differed among formulations rather than following a common temporal pattern. Roselló-containing formulations generally showed decreasing trajectories, whereas most Taiwan-containing formulations showed comparatively limited change or increasing syneresis during storage (Table 4).

The contrasting temporal patterns of Roselló- and Taiwan-containing formulations indicate that paste stability was associated not only with the proportion of elephant garlic but also with the *A. sativum* genotype used in the mixture. This interpretation is consistent with documented genotype- and species-dependent differences in S-alk(en)ylcysteine sulfoxide profiles in garlic and other *Allium* species [52,53], which may contribute to differences in technological behavior. Genotypic variation in physiological and biochemical responses related to water retention has also been reported in garlic and onion [54,55], although these studies involved intact plant tissues and therefore provide only indirect support for the present findings. Collectively, these findings support a formulation-specific basis for the differences in physical stability observed during storage.

### 3.3. Functional and Chemical Properties

The study results show that the antioxidant capacity of garlic paste varies significantly according to genotype and the proportion of garlic used in the paste, as shown in Table 5.

**Antioxidant capacity:** Antioxidant capacity differed significantly among formulations (*F*(8, 18) = 48.50, *p* < 0.001; Table 5). Among the mixtures, E_50_T_50_ showed the highest response and was statistically comparable with E_100_ and E_75_T_25_, whereas E_25_R_75_ showed the lowest antioxidant capacity. Mixture-model analysis showed that the E + T system exhibited a significant positive deviation from additivity (β_12_ = 19.0, *p* = 0.019), whereas the E + R system was consistent with additive behavior (β_12_ = −10.2, *p* = 0.455).

The significant positive deviation from additivity observed in the E + T system indicates that its antioxidant response could not be explained solely by the linear contributions of the pure components, whereas the E + R system remained consistent with additive behavior. This formulation-dependent response is consistent with previous reports showing substantial variation in antioxidant activity among garlic types, species, and cultivars [56,57,58]. Differences in garlic phytochemical composition, particularly in phenolic and organosulfur constituents, are also influenced by genetic background and pre- and postharvest conditions [32,59], while processing can markedly modify both composition and antioxidant activity [60]. Although these compositional differences provide a plausible context for the enhanced response observed in the E + T system, the compounds responsible were not quantified in the present study; therefore, the underlying mechanism cannot be established.

**Figure 3:**
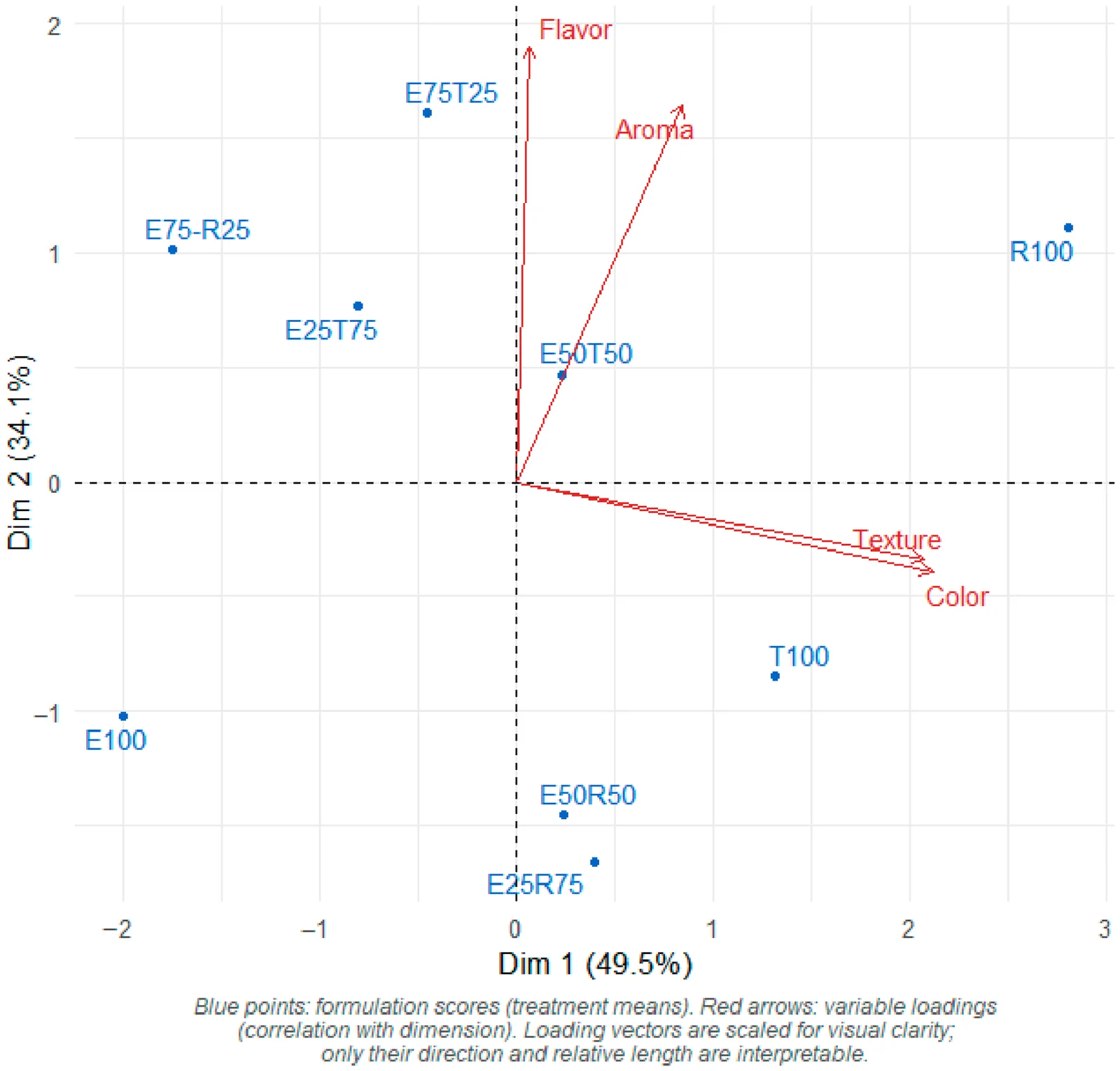
Principal component analysis (PCA) of sensory attributes and formulation scores of garlic pastes prepared with *Allium ampeloprasum* and *Allium sativum* genotypes.

**Sensory evaluation:** Descriptive sensory acceptance scores for aroma, color, texture, and flavor for each formulation are presented in Table S1. Principal component analysis (PCA) summarized 83.5% of the total variability in the mean sensory profiles through the first two dimensions (Figure 3). Dimension 1 (49.4%) was mainly associated with color and texture, which contributed 47.2% and 45.2% to this dimension, respectively. Dimension 2 (34.1%) was primarily associated with flavor and aroma, with contributions of 54.8% and 41.2%, respectively. Detailed variable correlations and contributions to the first two dimensions are provided in Table S2. Thus, sensory variation among formulations was structured along two complementary dimensions, one predominantly related to color and texture and the other to flavor and aroma. The distribution of formulations in the PCA space revealed distinct sensory profiles rather than a single overall acceptability gradient. Formulations differed in their positioning relative to these two sensory dimensions, indicating that different genotype combinations and mixture proportions were associated with different sensory profiles rather than one formulation consistently maximizing all evaluated attributes.

## 4. Conclusions

Combining *Allium ampeloprasum* with different *Allium sativum* genotypes enabled the development of garlic paste formulations with distinct physicochemical, technological, antioxidant, and sensory properties, with the response depending on both mixture proportion and the genetic material used. Mixture modeling showed that deviations from additivity were variable- and genotype-dependent rather than generalized across formulations. In particular, antioxidant capacity showed a significant positive deviation from additivity in the *A. ampeloprasum* + Taiwan 5 system, whereas syneresis exhibited strongly formulation-dependent temporal behavior during storage. These findings indicate that formulation performance was determined not only by component proportions but also by the specific garlic materials combined, highlighting the importance of raw-material selection in product development.

Sensory analysis further showed that the formulations differed along complementary dimensions mainly associated with color–texture and aroma–flavor, indicating that changes in mixture composition produced distinct sensory profiles rather than a uniform response across attributes. Overall, the results demonstrate that combining *A. ampeloprasum* and *A. sativum* can generate specific technological, antioxidant, and sensory responses depending on genotype and mixture proportion. Substantial proportions of *A. ampeloprasum* could be incorporated while maintaining technologically and sensorially relevant characteristics, supporting its potential as a complementary raw material for differentiated garlic-based products. However, commercial application should remain conditional on further validation of product safety, shelf stability, and formulation performance under industrially relevant conditions.

## 5. Study Limitations

Several limitations should be considered when interpreting the present results. First, water activity and microbiological stability were not evaluated; therefore, the physicochemical and rheological responses reported here should not be interpreted as evidence of product safety or established shelf life. In addition, the exact interval between processing and the nutritional, physicochemical, and antioxidant determinations conducted at room temperature was not systematically documented, limiting temporal interpretation of these measurements. Antioxidant capacity was assessed using a single DPPH-based assay, and individual phenolic and organosulfur compounds were not quantified; consequently, the mechanisms underlying the observed formulation-dependent antioxidant responses cannot be established. Likewise, the study characterized selected mixture proportions under a fixed base formulation containing the same levels of preservative and thickener across treatments and did not evaluate alternative preservation or structuring systems. Finally, the experimental design comprised three independent production batches per formulation and three predefined mixture proportions for each binary system; thus, the results characterize formulation responses within the evaluated experimental domain rather than defining a fully optimized commercial product. Further studies incorporating microbiological validation, water-activity measurements, complementary antioxidant assays, compositional and rheological characterization, and broader formulation optimization are required before industrial-scale recommendations can be made.

## Figures and Tables

**Table 1:** Biomass Proportions of Garlic Genotypes and Species Used to Produce the Pastes.

Genotype and Species	Garlic Proportion in the Paste *
*A. ampeloprasum* (*Elephant*)	E_100_
*A. sativum* Roselló 1	R_100_
*A. sativum* Taiwán 5	T_100_
Elephant:Roselló 1	E_75_R_25_
Elephant:Taiwán 5	E_75_T_25_
Elephant:Roselló 1	E_50_R_50_
Elephant:Taiwán 5	E_50_T_50_
Elephant:Roselló 1	E_25_R_75_
Elephant:Taiwán 5	E_25_T_75_

* The letter in each ratio corresponds to the genotype (E = Elephant, R = Roselló 1, and T = Taiwan 5), whereas the subscript value corresponds to the proportion of each garlic genotype in the paste.

**Table 2:** Protein, Fat, Ash, and Moisture Percentages in Garlic Pastes with Different Proportions of *Allium ampeloprasum* and *Allium sativum*.

Treatment *	Protein (%)	Fat (%)	Ash (%)	Moisture (%)
E_100_	3.56 ± 0.04 ab **	1.96 ± 0.01 f	13.96 ± 0.02 h	67.54 ± 0.26 c
R_100_	4.77 ± 0.02 e	1.73 ± 0.02 c	12.96 ± 0.02 e	62.72 ± 0.65 ab
T_100_	4.67 ± 0.01 de	2.18 ± 0.02 h	12.04 ± 0.02 b	61.10 ± 0.53 a
E_75_R_25_	3.64 ± 0.04 bc	1.33 ± 0.01 a	13.63 ± 0.02 f	63.68 ± 0.66 ab
E_75_T_25_	3.25 ± 0.02 a	1.94 ± 0.02 f	12.54 ± 0.02 d	65.23 ± 0.69 bc
E_50_R_50_	3.66 ± 0.02 bc	1.63 ± 0.02 b	12.24 ± 0.02 c	63.62 ± 0.64 ab
E_50_T_50_	3.97 ± 0.01 c	1.80 ± 0.02 d	13.86 ± 0.02 g	65.03 ± 0.80 bc
E_25_R_75_	3.94 ± 0.03 c	1.84 ± 0.01 e	11.89 ± 0.02 a	60.22 ± 0.52 a
E_25_T_75_	4.37 ± 0.01 d	2.03 ± 0.01 g	12.53 ± 0.02 d	63.70 ± 0.42 ab

* The letter in each ratio corresponds to the genotype (E = Elephant, R = Roselló 1, and T = Taiwan 5), whereas the subscript value corresponds to the proportion of each garlic genotype in the paste. ** Different letters within the same column indicate significant differences among treatments.

**Table 3:** Relationship among pH, Total Soluble Solids (TSS), and Color (*L*, *a*, and *b*) in Garlic Pastes with Different Proportions of *Allium ampeloprasum*.

Treatment *	pH	TSS (°Brix)	Color Parameters		
			*L*	*A*	*B*
E_100_	4.86 ± 0.19 a **	29.07 ± 0.06 a	47.00	−36.80	25.07
R_100_	5.68 ± 0.02 g	34.53 ± 0.06 f	82.03	−4.10	22.10
T_100_	5.60 ± 0.07 g	33.30 ± 0.20 e	79.77	−3.47	10.40
E_75_R_25_	5.06 ± 0.04 b	30.53 ± 0.12 b	54.13	−4.47	32.27
E_75_T_25_	5.09 ± 0.01 bc	32.87 ± 0.21 d	66.63	−22.50	28.67
E_50_R_50_	5.24 ± 0.17 cd	32.33 ± 0.06 c	68.00	−10.40	33.57
E_50_T_50_	5.29 ± 0.02 de	32.43 ± 0.15 c	84.30	−10.67	23.23
E_25_R_75_	5.56 ± 0.01 fg	32.83 ± 0.06 d	74.30	−17.83	32.67
E_25_T_75_	5.42 ± 0.01 ef	32.77 ± 0.06 d	72.97	−7.33	26.53

* The letter in each ratio corresponds to the genotype (E = Elephant, R = Roselló 1, and T = Taiwan 5), whereas the subscript value corresponds to the proportion of each garlic genotype in the paste. ** Different letters within the same column indicate significant differences among treatments.

**Table 4:** Syneresis of Garlic Pastes Formulated with Different Proportions of *Allium ampeloprasum* and *Allium sativum* during 21 Days of Storage.

Treatment *	Syneresis (%) During Storage			
	7 Days	14 Days	21 Days	Relative Change from Day 7 to Day 21 (%) **
E_100_	56.80 ± 0.20 g	44.53 ± 0.50 c	32.26 ± 1.10 c	43.21 ± 2.07
R_100_	43.70 ± 0.20 a	33.85 ± 0.04 a	23.99 ± 0.28 b	45.10 ± 0.89
T_100_	51.25 ± 0.25 cd	54.75 ± 0.05 de	58.25 ± 0.35 g	−13.66 ± 1.24
E_75_R_25_	53.64 ± 0.15 e	33.11 ± 0.07 a	12.57 ± 0.29 a	76.57 ± 0.61
E_75_T_25_	55.30 ± 0.20 f	54.37 ± 0.22 d	53.44 ± 0.34 e	3.36 ± 0.61
E_50_R_50_	52.20 ± 0.20 d	37.71 ± 0.21 b	23.23 ± 0.62 b	55.49 ± 1.36
E_50_T_50_	53.91 ± 0.20 e	55.47 ± 0.16 ef	57.03 ± 0.22 f	−5.80 ± 0.50
E_25_R_75_	49.15 ± 0.15 b	44.43 ± 0.43 c	39.71 ± 0.78 d	19.21 ± 1.52
E_25_T_75_	50.91 ± 0.20 c	56.44 ± 0.12 f	61.96 ± 0.44 h	−21.70 ± 1.34

* The letter in each treatment code identifies the garlic material (E = elephant garlic, R = Roselló 1, and T = Taiwan 5), whereas the numerical value indicates its proportion in the garlic component of the formulation. Values are means ± standard deviation of three independent production batches. Different lowercase letters within the same storage day indicate significant differences among formulations based on pairwise comparisons of estimated marginal means with Tukey adjustment (*p* < 0.05). ** Relative change from Day 7 to Day 21 was calculated for each independent batch as [(S7 − S21)/S7] × 100. Positive values indicate a decrease in syneresis, whereas negative values indicate an increase. Relative change values are descriptive; statistical inference for syneresis was based on the linear mixed-effects model.

**Table 5:** Antioxidant Capacity of Garlic Pastes with Different Proportions of *A. ampeloprasum* and *A. sativum*.

Treatment *	Antioxidant Capacity (μmolTE/100 g)
E_100_	38.96 ± 0.91 ab
R_100_	28.54 ± 1.75 c
T_100_	28.96 ± 0.53 c
E_75_R_25_	28.38 ± 1.00 c
E_75_T_25_	37.58 ± 2.06 ab
E_50_R_50_	37.29 ± 2.63 b
E_50_T_50_	41.71 ± 0.94 a
E_25_R_75_	23.14 ± 0.88 d
E_25_T_75_	31.46 ± 0.78 c

* The letter in each treatment code identifies the garlic material (E = elephant garlic, R = Roselló 1, and T = Taiwan 5), whereas the numerical value indicates its proportion in the garlic component of the formulation. Values are means ± standard deviation of three independent production batches. Different lowercase letters indicate significant differences among formulations according to Tukey’s HSD test (*p* < 0.05).

## Data Availability

The data presented in this study are available from the corresponding author upon reasonable request.

## Supplementary Materials

The following supporting information can be downloaded at: https://www.mdpi.com/article/10.3390/foods15183203/s1, Table S1: Descriptive statistics of sensory acceptance scores for the garlic paste formulations; Table S2: Correlations and contributions of sensory attributes to the first two PCA dimensions; Table S3: Quadratic Scheffé mixture-model interaction coefficients for response variables included in the manuscript.
