# Supplementary material for: Proximate, Physicochemical, Functional and Sensory Responses of Garlic Paste Formulations Combining Allium sativum and Allium ampeloprasum
Source: Foods. 2026 Sep 10;15(18):3203. doi: 10.3390/foods15183203 (PMC13605570; doi:10.3390/foods15183203)
Supplement: Supplementary file 1 [file foods-15-03203-s001.zip › foods-4515903-supplementary.pdf]

Supplementary Materials

Proximate, Physicochemical, Functional, and Sensory Responses of Garlic Paste Formulations Combining *Allium sativum* and *Allium ampeloprasum*

Table S1. Descriptive statistics of sensory acceptance scores for the garlic paste formulations.

Formulation	Aroma	Color	Texture	Flavor
E100	3.05 ± 1.21	3.37 ± 1.30	3.55 ± 1.13	2.95 ± 1.12
R100	3.55 ± 1.43	4.05 ± 1.37	4.00 ± 1.10	3.38 ± 1.11
T100	3.05 ± 1.29	3.93 ± 1.21	3.92 ± 1.17	3.27 ± 1.02
E75R25	3.23 ± 1.38	3.32 ± 1.24	3.57 ± 1.32	3.45 ± 0.98
E75T25	3.57 ± 0.99	3.60 ± 1.04	3.55 ± 0.73	3.29 ± 1.01
E50R50	3.20 ± 1.52	3.80 ± 1.22	3.72 ± 1.17	2.75 ± 1.02
E50T50	3.32 ± 1.11	3.65 ± 0.99	3.77 ± 1.16	3.28 ± 1.12
E25R75	3.17 ± 1.52	3.80 ± 1.25	3.77 ± 1.18	2.73 ± 1.02
E25T75	3.20 ± 1.30	3.60 ± 1.24	3.60 ± 1.12	3.48 ± 1.08

Note: Values are mean ± standard deviation (SD) of scores provided by 60 untrained consumers using a structured 5-point hedonic scale (1 = dislike extremely; 5 = like very much). No inferential grouping letters are shown because these values are presented descriptively.

Table S2. Correlations and contributions of sensory attributes to the first two PCA dimensions.

Sensory attribute	Correlation with Dim1	Contribution to Dim1 (%)	Correlation with Dim2	Contribution to Dim2 (%)
Aroma	0.3871	7.57	0.7487	41.13
Color	0.9660	47.16	−0.1786	2.34
Texture	0.9458	45.21	−0.1534	1.73
Flavor	0.0321	0.05	0.8643	54.81

Note: PCA was performed on standardized mean hedonic scores for aroma, color, texture, and flavor. Dim1 had an eigenvalue of 1.9786 and explained 49.47% of total variability; Dim2 had an eigenvalue of 1.3629 and explained 34.07%. Cumulative variance explained by Dim1 and Dim2 was 83.54%. Correlations indicate the direction and strength of association of each attribute with a dimension, whereas contributions indicate the percentage contribution of each attribute to that dimension.

Table S3. Quadratic Scheffé mixture-model interaction coefficients for response variables included in the manuscript.

Response variable	E + R β12	p value	Interpretation	E + T β12	p value	Interpretation
Protein (%)	−2.00	<0.001	Negative deviation	−1.05	0.195	Consistent with additivity
Fat (%)	0.22	0.426	Consistent with additivity	−2.13	<0.001	Negative deviation
Ash (%)	−4.40	0.001	Negative deviation	0.89	0.564	Consistent with additivity
Moisture (%)	−10.71	0.041	Negative deviation	1.96	0.688	Consistent with additivity
pH	0.015	0.957	Consistent with additivity	0.18	0.399	Consistent with additivity
TSS (°Brix)	0.95	0.209	Consistent with additivity	6.59	0.005	Positive deviation
Color L*	7.28	0.203	Consistent with additivity	62.48	0.001	Positive deviation
Color a*	44.23	<0.001	Positive deviation	33.56	<0.001	Positive deviation
Color b*	43.12	<0.001	Positive deviation	35.12	0.003	Positive deviation
Syneresis, Day 7 (%)	7.06	0.001	Positive deviation	−2.39	0.177	Consistent with additivity
Syneresis, Day 14 (%)	−4.33	0.746	Consistent with additivity	26.49	<0.001	Positive deviation
Syneresis, Day 21 (%)	−15.72	0.534	Consistent with additivity	55.36	<0.001	Positive deviation
DPPH-based antioxidant capacity (µmol TE/100 g)	−10.2	0.455	Consistent with additivity	19.0	0.019	Positive deviation

Note: Models were fitted separately for the *A. ampeloprasum* + Roselló 1 (E + R) and *A. ampeloprasum* + Taiwan 5 (E + T) binary systems using the individual observations from three independent production batches per formulation (N = 15 observations per binary system). The quadratic Scheffé model was $Y = \beta 1X1 + \beta 2X2 + \beta 12X1X2$. The β12 term quantifies deviation from the linear additive expectation. A statistically significant positive β12 indicates a positive deviation from additivity, a statistically significant negative β12 indicates a negative deviation, and a non-significant term indicates behavior consistent with additivity. The sign of β12 indicates mathematical direction and should not be interpreted universally as beneficial or detrimental; for syneresis, for example, lower values may represent greater physical stability.
